# Validation of the blood gas analyzer for pH measurements in IVF culture medium: Prevent suboptimal culture conditions

**DOI:** 10.1371/journal.pone.0206707

**Published:** 2018-11-12

**Authors:** Juan D. N. Diaz de Pool, Sjoerd A. A. Van Den Berg, Gonneke S. K. Pilgram, Bartholomeus E. P. B. Ballieux, Lucia A. J. Van Der Westerlaken

**Affiliations:** 1 Department of Gynecology, Leiden University Medical Center, Leiden, The Netherlands; 2 Department of Clinical Chemistry, Leiden University Medical Center, Leiden, The Netherlands; Central University of Tamil Nadu, INDIA

## Abstract

Measurement of pH in IVF-media using the blood gas analyzer (BGA) requires validation, because IVF-media is outside the intended scope of the BGA. To determine whether the Siemens Rapidpoint 500 BGA is suitable for pH measurements in IVF-media this study will validate the BGA and assess its accuracy. In this method comparison study, the pH of over three hundred IVF-media samples was measured with the BGA and a pH electrode (Hanna pH checker). The precision of both the BGA and the pH electrode were excellent (coefficient variation <1.4%). However, the closeness of agreement between measured values of both devices were not equivalent to each other in the tested IVF-media, showing 15% to 85% accordance between devices. The pH measured with the blood gas analyzer was also significantly higher in the tested media, compared to that measured by the pH electrode. One of the tested media did not reach its target pH when it was measured with the BGA, even at 9% CO_2_. The results show that the validated blood gas analyzer produces excellent results in terms of precision but not in terms of accuracy. Inaccurate measurement may lead to misinterpretation of results and consequently to suboptimal culture conditions. Therefore, each laboratory is encouraged to perform a validation of their BGA.

## Introduction

Culture conditions play an important role in the development of oocytes and embryos [1]. Factors that may contribute to the culture condition and consequently to the development of the oocytes and embryos are mainly media composition [2], osmolality [3] temperature [4–8] and pH [9–11]. The last two factors can be controlled within the laboratory. As the pH is measured on a logarithmic scale, minor changes in pH reflect large changes in H^+^ concentration [1]: a difference in pH of 0.3 units reflects a 99.5% change in H^+^ concentration. Human oocytes and embryos are sensitive to extracellular pH (pH_e_), although these cells have active transport mechanisms for the regulation of internal pH (pH_i_) [11–16]. Combating changes in pH_i_ caused by pH_e_ may result in diversion of energy from vital cellular developmental functions. Denudated oocytes and cryopreserved embryos lack pH_i_ regulatory mechanisms, which makes them completely depended of the pH_e_ as shown in studies with animals [15,17–21]. Therefore, measurement and management of pH in culture media are important in order to prevent improper culture conditions and consequently detrimental effects on oocyte maturation [1,12,22,23] and embryo development [1,12,13,15,17,24].

For the measurement of pH in IVF culture medium it is important that the measuring method gives accurate results. The blood gas analyzer (BGA) is routinely used to measure pH in culture media. Although method validation is required when the BGA is being used outside its intended scope, no technical validation of the BGA for measurement of IVF-media has been published. Furthermore, it has been recognized that not all analyzers are equal and that it is prudent to validate the accuracy before use [25,26]. The BGA is recommended based on its accuracy [1,25], however, it is unclear how the accuracy is determined. This is important, because the definition and method by which it is determined may differ between studies. Moreover the terms “accuracy” and “precision” are used interchangeably in literature, while these terms have conflicting meaning [27]. Precise results only represent the imprecision of the method, but don’t represent the closeness of agreement between the true pH value and the value obtained with the BGA.

Without a proper validation it remains unknown whether the measured pH represents the actual pH. Therefore, this study will evaluate the accuracy of pH measurements in IVF culture media using a BGA. In order to assess the accuracy of the BGA for pH measurements in IVF culture media, the BGA will be validated by a state of the art method comparison with a portable pH electrode. The accuracy consists both of trueness and precision of the measurement [27,28]. Precision will be determined in both methods by assessing the closeness of the measured values to each other. Trueness will be assessed by the closeness of agreement between measured values of the BGA and the values obtained from the pH electrode. The CO_2_% at which the target pH is reached will be determined by measurement of the pH in the culture media at different CO_2_%.

## Materials and methods

### Ethical approval

This study was performed without patients or patient data; therefore, the medical ethical committee of the Leiden University Medical Center stated that their approval is not required for the research to be undertaken. This validation is based on guidelines from the clinical and laboratory standards institute (CLSI).

### Measuring methods and calibration

pH was measured using a blood gas analyzer (BGA) (Siemens RAPIDPoint 500 (SRP); Siemens Healthcare, Sudbury, UK) and a portable pH electrode (pH checker HI98103, Hanna Instruments, Nieuwegein, The Netherlands). The SRP system has an on-board calibration module, performing a single point calibration every 30 minutes and a 2 point calibration every 2 hours. The SRP pre-warms the samples and keeps them at a constant temperature (37°C) during measurement. In order to adjust the output to reflect the “true” value, the pH electrode was calibrated with certified calibration buffers obtained from Radiometer (pH 7, cat. No. S11M004, lot no. C02337 and pH 9, cat. No. S11M006, lot no. C02270). These certified standards are traceable to the national institute for standards and technology (NIST) and are internationally recognized and considered as the ultimate authority. Prior to calibration of the pH electrode, both the electrode and the calibration buffers were stored in 15 ml tubes (Falcon, cat. No. 352095) and pre-warmed overnight inside the incubator (Heracell 240, Germany). The calibration took place inside the incubator. To avoid influence of CO_2_ on the calibration buffers, all tubes containing calibration buffer were pre-warmed with closed caps. The electrode was stored in NaCl 0.9% solution, preventing direct influence of the CO_2_ on the electrode. The pH readings obtained with the pH electrode were adjusted for temperature (37°C) according to the manufacturer’s recommendations (6.97 and 9.09 for calibration buffer pH 7 and pH 9 respectively) each day prior to measurement. Drift was verified each day by measuring the pH of the calibration buffers before and at the end of the measurements. To assure that the CO_2_ level in the incubator was accurate, the CO_2_ was measured with calibrated CO_2_ probes. The CO_2_ measuring devices used in this study are calibrated yearly by the manufacturer (Vaisala, Finland). The frequency for conducting calibration of the CO_2_ probes is based on manufacturers recommendation and historic information of previous calibrations.

### Material

The pH_e_ was measured in the sequential culture media from Origio (ORIGIO Sequential Series, Charlottesville, USA) consisting of three different media (Sequential Fert, cat. No. 83030060, Sequential Cleav, cat. No. 83040010 and Sequential Blast, cat. No. 83060010). Each medium has its own recommended pH (Fert pH 7.35, Cleav pH 7.20 and Blast pH 7.30), defined as target pH ±0.1pH unit. Prior to pH measurements, the culture media were incubated overnight in 5 ml tubes (Falcon, cat. No. 352058) to ascertain pH equilibrium. CO_2_% and temperature (°C) inside the incubator was set to the manufacturers specifications (5–6% CO_2_ and 37°C).

### Accuracy: Trueness, reproducibility and repeatability

Accuracy was determined by assessing repeatability, reproducibility and trueness of the measuring methods. Trueness of the pH electrode was assessed by calibrating the pH electrode using calibration buffers that are traceable to the national institute for standards and technology (NIST) and confirming that drift never exceeded 0.1 pH units at various CO_2_%, ranging from 5% to 9% with 1% intervals. Trueness of the BGA was determined by assessing the closeness of agreement between the pH electrode and the BGA by duplicate measurement of all media at various CO_2_%, ranging from 5% to 9% with 0.5% intervals. Additionally, the optimal CO_2_% was determined for each culture medium. Precision, i.e. closeness of measured values to each other, can be subdivided in repeatability and reproducibility. At the optimal CO_2_% (CO_2_% at which target pH is reached), repeatability was estimated for both instruments by tenfold measurement for each of the culture media. Reproducibility was also estimated at the optimal CO_2_% for both instruments in all media by triplicate measurement, trice daily for a period of five days.

### pH measurement

BGA measurements were performed at the central laboratory of the Leiden University Medical Center. To ascertain temperature and pH stability, all tubes containing medium were closed inside the incubator before removing them from the incubator. Tubes destined for BGA measurements were transported to the central laboratory in a temperature controlled (37°C) transport box (MDT, cat. No. TC-07UG-12) and the measurements were performed by one operator. Upon arrival (time to arrival: 6 minutes), samples were aspirated within a 1 ml syringe and immediately plugged into the BGA for the pH measurement. Measurement of the culture medium with the calibrated pre-warmed pH electrode was performed inside the same incubator as where the culture media were incubated. The door of the incubator remained closed during the pH measurement and the gas tight inner glass door of the incubator allowed pH reading without disturbing the inner atmosphere.

### Data analysis

Data analysis was performed using SPSS (IBM, version 23). Trueness of the BGA was determined by the comparison of the results, between the two pH measuring methods, using an allowable Total Error (TEa) of 0.1 pH unit. The TEa is used in comparability testing to ensure that the measured values of the methods are similar and that they can be used interchangeably without causing clinical error. Precision was assessed by the calculation of coefficient of variation (CV) of the repeatability and the reproducibility. The predefined allowable CV was 1.4%, which is equivalent to a deviation of approximately 0.1 pH unit from the target pH.

## Results

### Recommended CO_2_%

The CO_2_% recommended by the manufacturer is 5 to 6%. Table 1 shows the average of duplicate pH measurements in medium 1–3 at 5% and 6 CO_2_%, using the pH electrode and the BGA. These data show that the measured pH was outside the manufacturers recommended specification of CO_2_% (5–6% CO_2_).

**Table 1:** Measured pH in three different culture media, using the pH electrode and the blood gas analyzer at recommended CO_2_%.

**Recommended CO**_**2**_**%**	**Medium**	**Target pH**	**Measured pH**	
			pH electrode	Blood gas analyzer
5%	1	7.35	7.57^a^	7.54^a^
	2	7.2	7.32^a^	7.42^a^
	3	7.3	7.43^a^	7.46^a^
6%	1	7.35	7.34	7.56^a^
	2	7.2	7.29	7.44^a^
	3	7.3	7.35	7.48^a^

^a^ pH >0.1pH units outside the target pH

### Optimal CO_2_% using pH electrode

**Fig 1:**
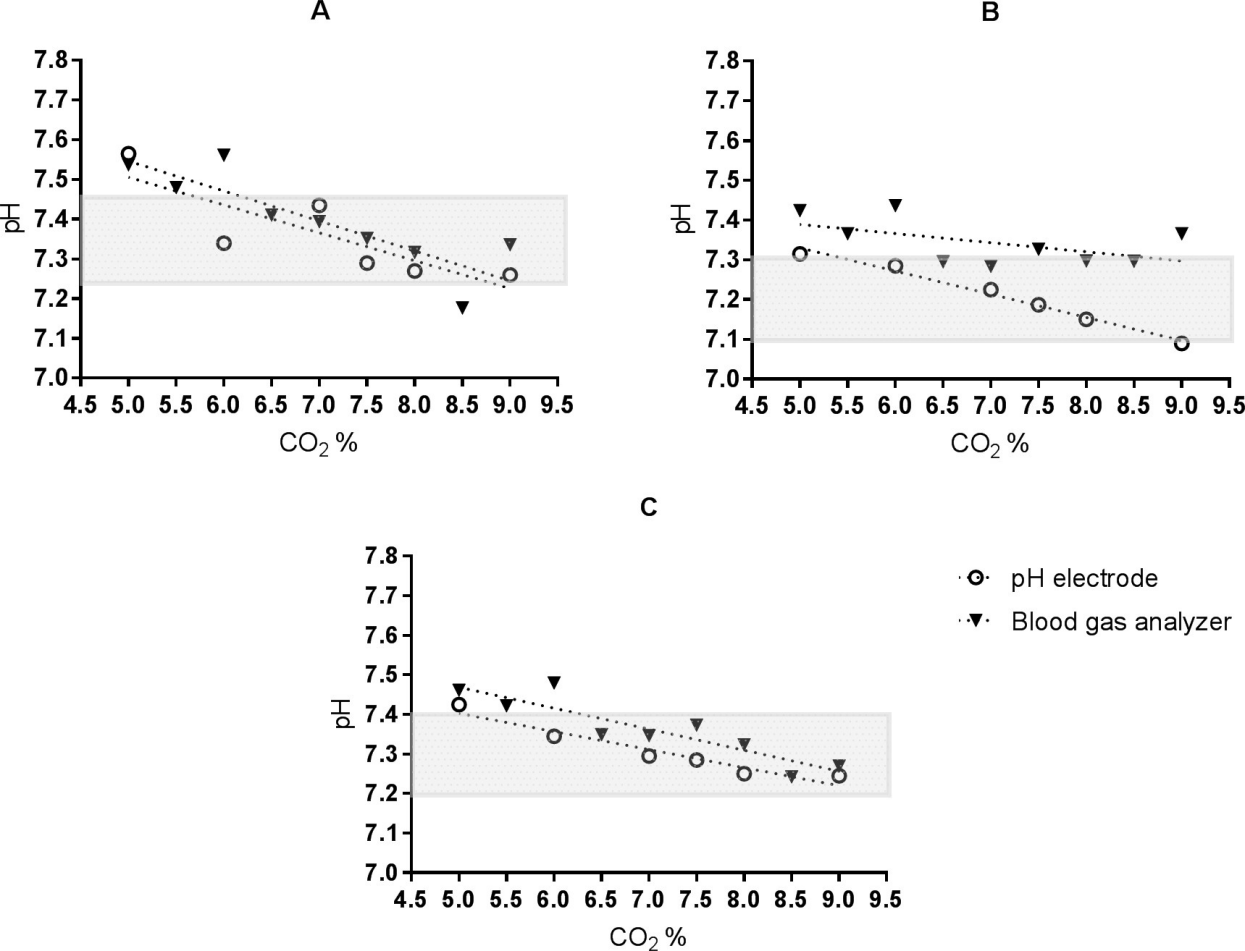
Response of pH to different CO_2_ levels. The relation between pH and CO_2_% is linear for all media when it is measured with the pH electrode. A: medium 1, B: medium 2, C: medium 3.

To identify the optimal CO_2_% (CO_2_% at which pH reaches the target level) for all media, we titrated CO_2_% inside the incubator. The relation between pH and CO_2_%, measured with the pH electrode, was linear for all media and showed a decrease in pH with the increase of CO_2_% (Fig 1). There was a statistical significant correlation in medium 1 (r_electrode_ = 0.84, P<0.05), medium 2 (r_electrode_ = 0.98, P<0.05) and medium 3 (r_electrode_ = 0.96, P<0.05) between CO_2_ and pH. Optimal CO_2_ values were 7.1%, 7.3% and 7.2% for medium 1 to 3, respectively. A CO_2_% within the range 5.7% to 8.4% resulted in a pH within the manufacturers recommended pH, defined as the target pH ±0.1pH unit, in all culture media when measured using the pH electrode. This shows that the lower limit of the recommended CO_2_ (5%) by the manufacturer is too low to reach the target pH.

### Optimal CO_2_% using blood gas analyzer

Based on the BGA measurements, the target pH of the IVF-media were reached at higher CO_2_% compared to the measurement of the pH electrode (Fig 1). Based on the linear regression lines shown in Fig 1, there was a decrease in pH with the increase of CO_2_% in both measurement methods and in all media. There was a statistical significant strong correlation in medium 1 (r_BGA_ = 0.86, P<0.05) and medium 3 (r_BGA_ = 0.90, P<0.05) between CO_2_ and pH when this relation was measured with the BGA. Medium 2 had a weak correlation (r_BGA_ = 0.54, P>0.05). Optimal CO_2_ values were 7.6%, 13.0% and 8.4% for medium 1 to 3, respectively. A CO_2_% of 8.4 to 8.9% resulted in the manufacturers recommended target pH ±0.1pH unit in all culture media when measured using the BGA. The linear regression lines of medium 1 (Fig 1A) and 3 (Fig 1C) indicates fixed bias between methods. The regression lines of medium 2 (Fig 1B) indicates proportional bias between methods. Medium 2 did not reach its target pH when using the BGA, even at 9% CO_2_.

### Trueness

**Fig 2:**
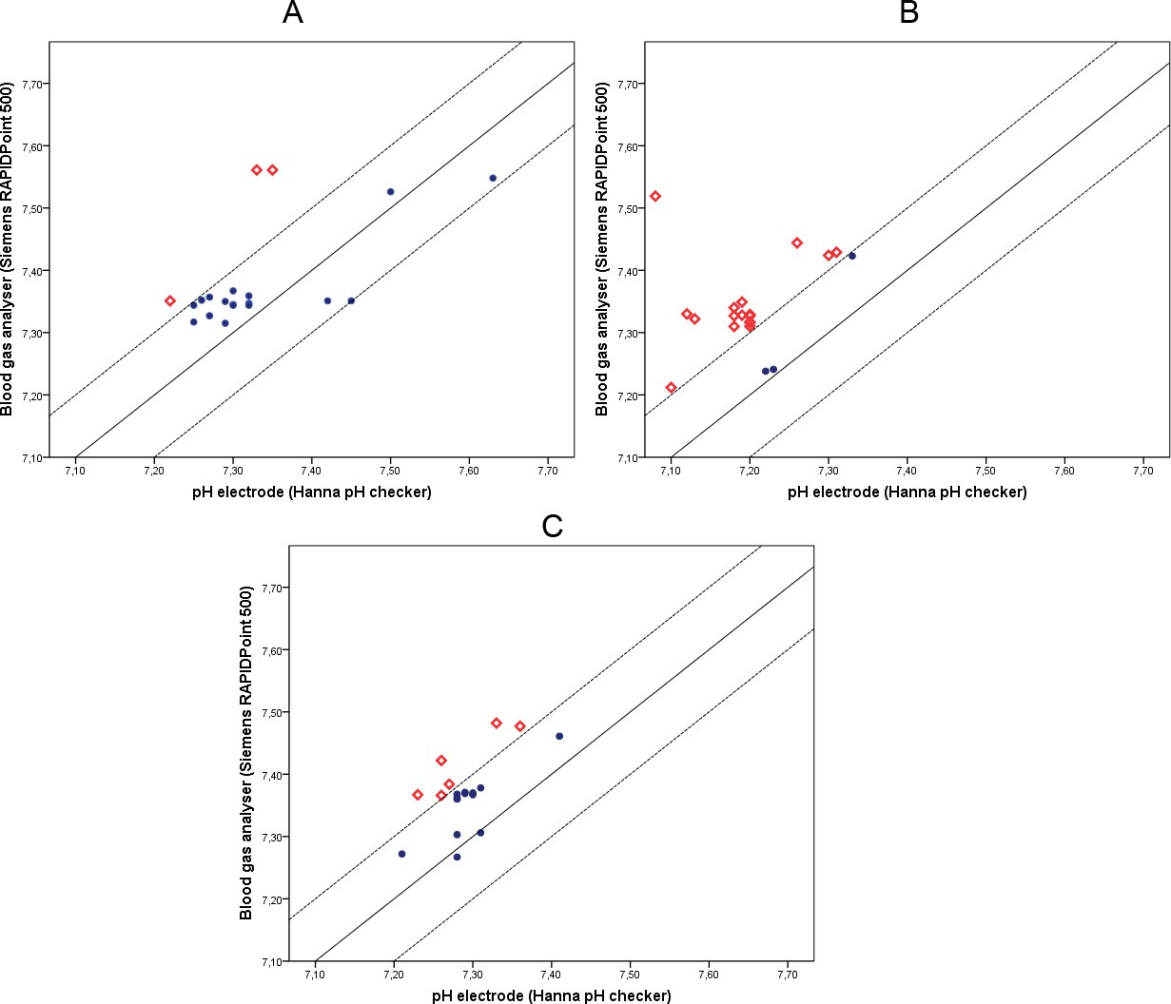
Closeness of agreement between pH electrode and blood gas analyzer (trueness). (●) Measurements that are within predefined allowable total error of 0.1 pH unit. (◊) Measurements that are outside predefined allowable total error of 0.1 pH unit in the three tested media. A: medium 1, B: medium 2, C: medium 3.

Calibrating the pH electrode with buffers that are traceable to the national institute for standards and technology (NIST) and confirming that drift never exceeded 0.1 pH unit at the different levels of CO_2_, proves that the measured values of the pH electrode are true. Trueness of the BGA was determined by method comparison. The results of the method comparison by duplicate measurement of all media at different CO_2_% are shown in Fig 2. Methods were equivalent within allowable total error (0.1 pH unit) for 17 of the 20 (85%) measurements in medium 1, for 3 of the 20 (15%) measurements in medium 2 and for 13 of the 19 (68.4%) measurements in medium 3 (Fig 2). Mean pH as measured using the BGA was significantly higher in all media compared to the pH electrode (P<0.01).

### Precision: Reproducibility and repeatability

Based on the results the CO_2_ level was adjusted to 7.5%, which is near the concentration to reach the target pH in all media according to the pH electrode. Mean pH at this CO_2_ concentration was 7.29 ± 0.03, 7.19 ± 0.02 and 7.29 ± 0.03, for medium 1 to 3, respectively, when measured using the pH electrode and 7.35 ± 0.01, 7.33 ± 0.01 and 7.37 ± 0.02 according to the BGA. Precision was determined by repeatability and reproducibility at the CO_2_ of 7.5%. The predefined allowable CV was 1.4%, which is equivalent to a deviation of approximately 0.1 pH unit from the target pH. The calculated CV of the repeatability was 0.4%, 0.3% and 0.2% for medium 1 to 3, respectively using the pH electrode and 0.1%, 0.2%, 0.2% using the BGA. Reproducibility was 1.1%, 1.2% and 1.1% for medium 1 to 3, respectively using the pH electrode and 0.9%, 0.4% and 0.5% using the BGA. This indicates for the repeatability and reproducibility a deviation of less than the predefined allowable CV of 1.4%, meaning a deviation of less than 0.1 pH units.

## Discussion

This study addresses the importance of determining the accuracy of the blood gas analyzer (BGA) for pH measurement in IVF-media. The main difference between the BGA (Siemens RAPIDPoint 500 (SRP)) and the pH electrode (pH checker HI98103, Hanna Instruments) for performing measurements in IVF-media is that the BGA is used outside its intended scope. In Fact, the BGA applies an algorithm designed for blood fluid (arterial blood, capillary venous blood and venous blood) on the IVF-media. In contrast to the BGA, the calibration buffers used for the pH electrode meets the criteria for metrological traceability, indicating that the measurement results can be related to an internationally traceable reference. The results concerning trueness, repeatability and reproducibility in combination with the internationally traceable buffers, show the high level of accuracy of the pH electrode. The BGA on the other hand, has an on-board calibration module, but has no independent trueness verifier. Commonly, proficiency testing for BGAs is assessed by external quality control based on consensus instead of internationally traceable references. Therefore trueness of the BGA for measurements in IVF-media can only be performed by method comparison.

Our results show that the BGA produces excellent results in terms of precision (CV_repeatability, reproducibility_ <1.4%). In terms of trueness, the method comparison test (Fig 2) shows a high level of agreement (LA) in medium 1 (LA = 85%) and a low level of agreement in medium 2 (LA = 15%) and 3 (LA = 68.4%). Given that the international accepted definition of accuracy is described as a combination of trueness and precision [29–31], the tested BGA is not accurate for our IVF-media.

In our study, we used a CO_2_ response curve to determine accuracy of the pH measuring methods, not only at one specific CO_2_%, but also within the whole operating range. It should be investigated how the measuring methods behave in a broad CO_2_% range, in order to evaluate whether there is fixed bias or proportional bias between the methods. Determining trueness at only one specific point can generate invalid results, as one would correct for the observed difference between methods, based on one point, while this correction is not allowed if there is proportional bias. Results of the pH measurement in medium 2 with the BGA show the importance of this approach. The response curve of medium 2 shows clearly proportional bias (Fig 1B). Furthermore, the response curve of medium 2, measured with the BGA, shows a low correlation (r_BGA_ = 0.54, P>0.05) between CO_2_ and pH in medium 2 (Fig 1B). The pH of medium 2, measured with the BGA never reached the target pH, even at CO_2_ of 9%. From this we conclude that the BGA is not suitable for the pH measurement of medium 2.

The fixed bias seen in medium 1 and 3 could originate from analytical or pre-analytical errors. Analytical error was minimized by having the measurement performed by one operator. Pre-analytical errors were avoided by incubation of all media in the same incubator, closing all tubes destined for BGA measurement inside the incubator, transportation of these samples to the BGA in a temperature controlled transport box and measurement of the pH inside the incubator with the pH electrode. The high pH values obtained with the BGA could theoretically be the result of evaporation of CO_2_ during handling of the sample. In this case you would expect a fixed bias, instead of the proportional bias seen in medium 2. This proportional bias can originate from interfering media-specific-components in the IVF-medium on the BGA. The main problem for studying the effects of media-specific-components is that detailed descriptions of the media composition are undisclosed and that IVF-media contains media-specific-components that may interfere with the BGA.

Although it is clear that the BGA is not suitable for pH measurements in our IVF setting, the high level of disagreement between the recommended optimal CO_2_ by the manufacturer (5–6%) and the optimal CO_2_ level (~7.2%) determined in this study remains unclear. Discrepancies in reports between CO_2_ sensors have been described, showing different CO_2_ readings when the same CO_2_ concentration was measured [1]. In addition, the type of lab ware and even altitude can influence the CO_2_ gas exchange and consequently the pH of the culture medium [1]. Managing pH by CO_2_ monitoring without pH measurement is therefore unreliable.

The need to specifically tailor atmospheric CO_2_ to optimize culture medium pH has been recognized [25]. To ascertain that the correct pH is reached, one must choose a method to perform this check. In this respect, it is important to realize that not all BGAs are equal with respect to standardization and calibration [32,33]. Retrospective analysis of Dutch external quality control data (April 2016 –October 2016) revealed that, overall, Siemens BGAs are slightly positively biased when compared to Radiometer BGAs, but negatively biased when compared to Instrument Laboratory and Abbot BGAs. The magnitude of this difference is dependent on the absolute pH value but differences may exceed 0.1 pH unit. It is therefore important to assess what method was used to determine the optimal pH of the culture media and to adjust the results of the BGA to that method.

This study concerns a limited technical validation, because the research question was already answered at an early stage of the validation. Therefore measurement characteristics e.g. linearity, detection limit and range of measurement were not further investigated. Furthermore the pH electrode (pH checker HI98103, Hanna Instruments) used in this study is no longer recommended, because in the new model it is no longer possible to adjust the pH manually in order to correct the pH for temperature. A pH electrode that can be adjusted manually or automatically for temperature and with which you can measure within the incubator is advised.

In summary, we present a validation of the BGA (Siemens RAPIDPoint 500 (SRP)) for pH measurement in our IVF culture media. We show that the SRP produces excellent results in terms of precision, but that the pH measurements with this BGA are not always equivalent to the pH electrode. The need for validation is reinforced by the fact that the recommended CO_2_% deviates from the optimal CO_2_% determined in our study. Therefore, each laboratory is encouraged to perform a validation of the BGA being used in their laboratory. Small errors in report can lead to wrong culture conditions for oocytes and embryos.
